# Portable Dynamic Chest Radiography: Literature Review and Potential Bedside Applications

**DOI:** 10.3390/medsci12010010

**Published:** 2024-02-07

**Authors:** Maurizio Cè, Giancarlo Oliva, Francesca Lucrezia Rabaiotti, Laura Macrì, Sharon Zollo, Alessandro Aquila, Michaela Cellina

**Affiliations:** 1 Postgraduation School in Radiodiagnostics, Università degli Studi di Milano, Via Festa del Perdono 7, 20122 Milan, Italy; francesca.rabaiotti@unimi.it (F.L.R.); laura.macri@gmail.com (L.M.); alessandro.aquila@unimi.it (A.A.); 2 Radiology Department, Fatebenefratelli Hospital, ASST Fatebenefratelli Sacco, 20121 Milan, Italy; linforisonanza@gmail.com (G.O.); michaela.cellina@asst-fbf-sacco.it (M.C.); 3 Konica Minolta Business Solutions Europe GmbH, Capellalaan 65, 2132 JL Hoofddorp, The Netherlands; sharon.zollo@konicaminolta.eu

**Keywords:** dynamic digital radiography, diaphragmatic dysfunction, dynamic chest X-ray, diaphragm mobility, dynamic imaging

## Abstract

Dynamic digital radiography (DDR) is a high-resolution radiographic imaging technique using pulsed X-ray emission to acquire a multiframe cine-loop of the target anatomical area. The first DDR technology was orthostatic chest acquisitions, but new portable equipment that can be positioned at the patient’s bedside was recently released, significantly expanding its potential applications, particularly in chest examination. It provides anatomical and functional information on the motion of different anatomical structures, such as the lungs, pleura, rib cage, and trachea. Native images can be further analyzed with dedicated post-processing software to extract quantitative parameters, including diaphragm motility, automatically projected lung area and area changing rate, a colorimetric map of the signal value change related to respiration and motility, and lung perfusion. The dynamic diagnostic information along with the significant advantages of this technique in terms of portability, versatility, and cost-effectiveness represents a potential game changer for radiological diagnosis and monitoring at the patient’s bedside. DDR has several applications in daily clinical practice, and in this narrative review, we will focus on chest imaging, which is the main application explored to date in the literature. However, studies are still needed to understand deeply the clinical impact of this method.

## 1. Introduction

Dynamic digital radiography (DDR) is an X-ray imaging technology consisting of a fast-paced sequential acquisition of multiple X-ray scans further reproduced as a cine loop [1]. This technique allows for the observation and movement analysis of anatomical structures in different physiological and pathological conditions, providing functional and clinical information that cannot be extrapolated from static imaging.

Chest imaging examinations are often requested in hospitalized and traumatized patients, who present frequent mobilization difficulties.

Portable X-ray machines are useful tools for the diagnosis and monitoring of patients whose transfer to the hospital radiology department may be a challenging obstacle and represent the most convenient and cost-effective standard choice for the routine monitoring of patients with reduced mobility or in intensive therapy units [2]. However, traditional radiographies have several limitations since they provide only static morphological information.

First developed in the 2000s [3], DDR technology has gained increasing popularity in the last years and expanded its fields of application thanks to the now substantial literature in standing patients showing its advantages compared with other imaging modalities, including speed, ease of use, versatility, and low-dose radiation [1].

The first developed equipment was not transportable, thus limiting the widespread adoption in the hospital setting. However, recent technological improvements have expanded its range of clinical applications, adding the functional value of dynamic imaging to the versatility of portable equipment with wireless dynamic technology.

In this review, we will present the main clinical applications of DDR in studies of the chest, including lung ventilation and diaphragmatic motility, according to the available literature in orthostatic patients. Furthermore, we will discuss potential clinical applications of the newly released portable version, starting from the experience in our center.

## 2. The Equipment

**Figure 1:**
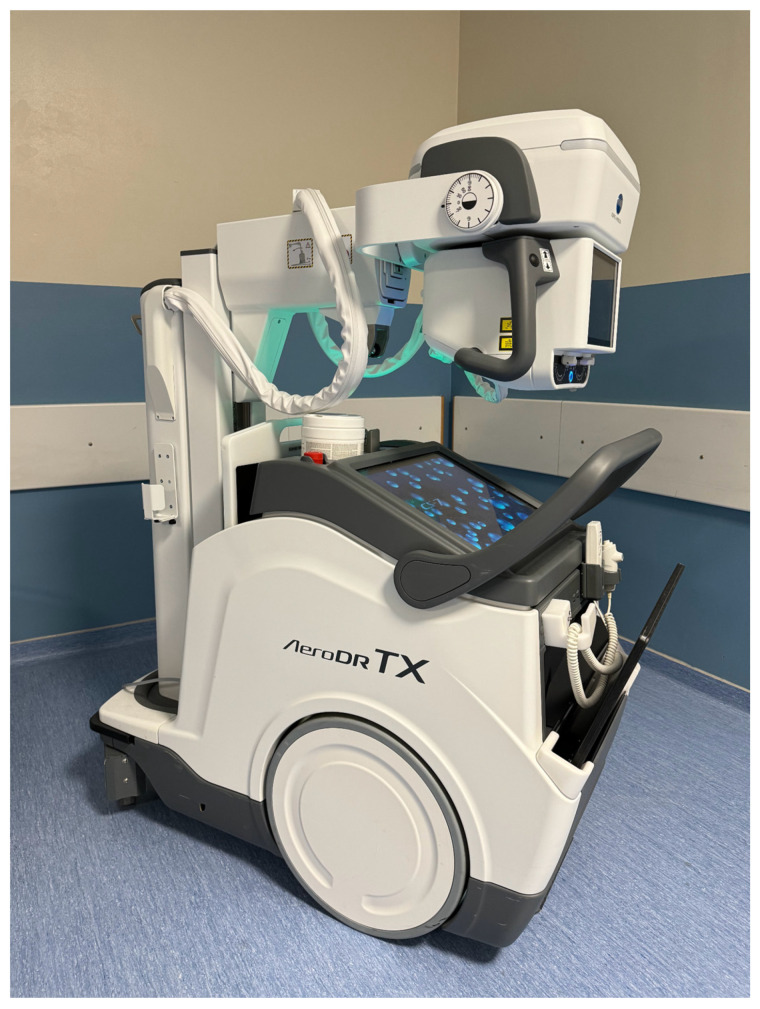
Mobile DDR equipment AeroDR TX m01 (manufactured by Sedecal, Madrid, Spain; distributed by Konica Minolta Business Solutions Europe GmbH, Hoofddorp, The Netherlands).

The last released equipment is a mobile device, the AeroDR TX m01 (manufactured by Sedecal, Madrid, Spain; distributed by Konica Minolta Business Solutions Europe GmbH, Hoofddorp, The Netherlands), paired with the AeroDR3 HD2 detector (Konica Minolta, Inc., Tokyo, Japan) that we use in our Institution. It has a footprint identical to that of a standard mobile portable X-ray machine and can be easily transported to the patient’s bed, significantly expanding the range of applications in different clinical settings (Figure 1).

**Figure 2:**
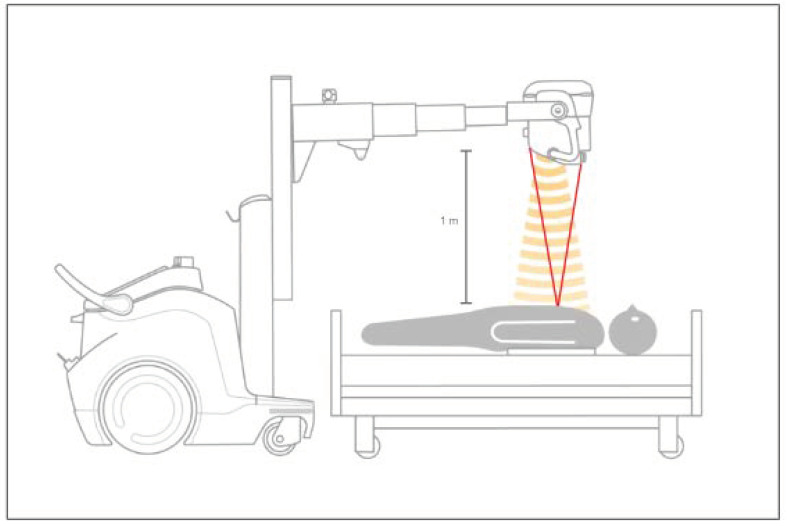
Standard configuration of the DDR equipment for dynamic image acquisition in a bedridden patient (courtesy of Konica Minolta, Inc., Tokyo, Japan). The X-ray emission is performed in a pulsatile way to reduce radiation exposure to the patient. The panel should be placed by the radiologist technician below the patient’s chest. The proper patient positioning with respect to the radiation source is achieved with the use of laser guidance provided by the equipment.

This compact device can be easily moved to different hospital wards (Figure 2).

The DDR execution is quick, even if takes a little longer than a standard X-ray [1]. The acquisition procedure is similar to that of standard chest radiography, which requires only some precautions for the optimal selection of acquisition parameters and patient positioning (Figure 2). In our experience, these simple aspects can be easily mastered by radiology technicians with rapid dedicated training.

Native images (static or dynamic) are directly sent to the RIS-PACS system via the WI-FI connection and to the dedicated DI-X1 workstation (Konica Minolta, Inc., Tokyo, Japan) for advanced post-processing to enable a precise quantitative assessment of organ kinetics and to conduct an in-depth functional analysis of pulmonary motion and ventilation. DDR is a cost-effective technology compared with other chest imaging methods like Computed Tomography (CT), scintigraphy, Magnetic Resonance, and fluoroscopic examination systems [1,4].

The DRR system uses a pulsed synchronized X-ray generator and a dynamic flat panel detector (AeroDR3 HD2, (Konica Minolta, Inc., Tokyo, Japan)). This X-ray emission generates serial fast-paced digital images that capture the anatomy in motion using a low radiation exposure. 

DDR provides sequential images of the entire lung with high temporal resolution (15 frames per second [fps]) during breathing in the standing, sitting, and supine positions [1,4]. The pixel size, matrix, and overall image area of the flat panel are 388 × 388 microns, 1024 × 768, and 40 × 30 cm, respectively. The standard acquisition time for the chest has a duration of 10–15 s, which corresponds to approximately 3–5 complete respiratory cycles in a normal patient but can be reduced if needed. The examination can be acquired in the tidal or forced respiratory cycle, depending on the focus of the examination. Shorter acquisitions (6 s) in breath-holding are used for the cardiac phase and pulmonary vessel perfusion analysis [5].

In recent years, several applications of DDR have been explored: chest imaging has attracted the greatest efforts of researchers due to the great amount of information that can be obtained from dynamic acquisitions, including the movement of the diaphragm, thoracic wall, lungs, and mediastinal structures.

**Figure 3:**
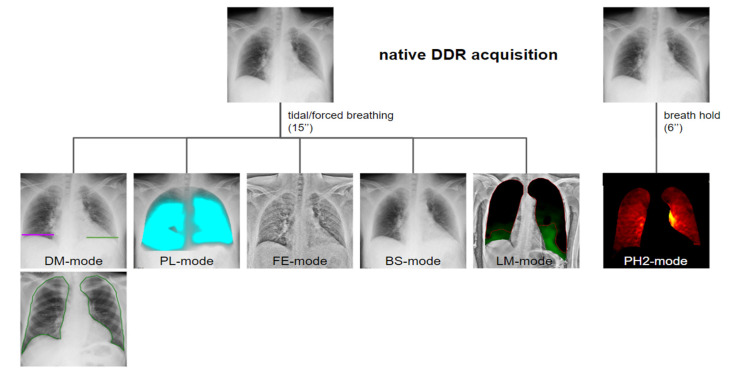
Different post-processing modes obtained from native DDR images. The perfusion study mode (PH2 mode) requires an acquisition of 6 s with breathing stopped. For all other modalities, the acquisition occurs with tidal or forced breathing (depending on clinical needs), and the standard duration is 15 s. DM = diaphragm motion; FE = frequency enhanced; PH2 = “pixel value change—high-frequency processing”; BS = bone suppression; LM = lung motion’; PL (reference frame ratio circulation) = “pixel value change—low-frequency processing”.

By post-processing standard sequential images, several additional reformations including bone suppression images, frequency-enhanced images (for soft tissue analysis), and ventilation and perfusion maps can be obtained. Furthermore, quantitative parameters such as diaphragmatic mobility, lung area, and the respective variations during the respiratory cycle are automatically calculated [4] (Figure 3 and Table 1).

In this review, we provide an overview of the existing literature on thoracic DDR performed in orthostatic patients and discuss some further perspectives and advantages of the potential bedside applications of the portable equipment.

## 3. Diaphragm Motion Analysis

The diaphragm is a dome-shaped musculotendinous structure located between the chest and abdominal cavity and is the primary inspiratory muscle. Its contraction determines a lower pleural pressure, which favors lung expansion during the inspiratory phase and the filling of gasses in the airways, while its relaxation increases thoracic pressure, allowing for the expiratory phase and the release of gasses into the air from the airways [4].

Diaphragmatic dysfunction is a challenging diagnosis that might be accompanied by mild clinical signs and can end in ventilatory failure even in the absence of any pathological abnormality of the lung parenchyma [6].

Few imaging modalities are available to investigate diaphragm mobility, and standard chest radiography remains the primary imaging tool for suspected diaphragmatic impairment. However, it is severely limited by being a two-dimensional static acquisition and provides only information on the diaphragm position [7].

Ultrasound, thanks to its non-invasiveness, can be used to study diaphragmatic mobility. However, it is limited by operator-dependency and poor visualization of the deep diaphragm areas [8,9].

**Figure 4:**
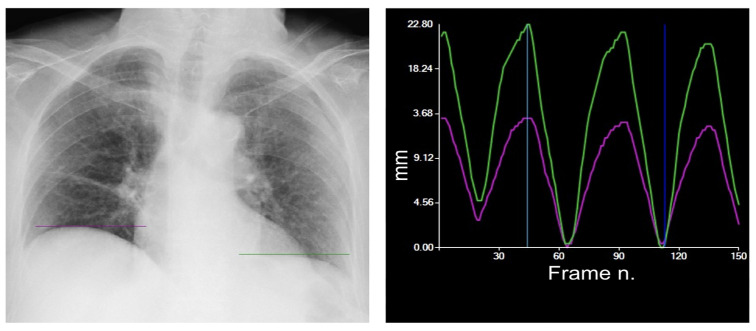
Typical tidal breathing pattern. The green line represents the tracking of the left diaphragm, whereas the purple line tracks the right diaphragm. The green curve represents the movement in millimeters over time, with a maximum peak during the exhale and a minimum peak during the inhale. The purple curve represents the right diaphragm. The most common tidal breathing pattern consists of a higher motion of the left diaphragm. The system automatically identifies the moment of maximum inspiration within the cycle (dark blue vertical line). The light blue vertical line can be moved along the graph to select a specific frame to view (in this case the point of maximum exhalation, on the left).

Native DDR images can be post-processed via the dedicated DM mode for the evaluation of diaphragmatic motion. Specifically, user-specified apical and diaphragmatic regions are tracked by combining edge extraction processing and pattern matching. The highest point of each diaphragm is tracked in a motion–time graph, where time is represented by the frame number. The maximum excursion for each hemidiaphragm is calculated and expressed in millimeters and the peak movement speed of each hemidiaphragm [4] (Figure 4).

Thanks to this modality, the DDR represents a valid tool in the case of suspected diaphragmatic dysfunction as it provides functional, structural, and morphological information in different conditions such as tidal, forced breathing, or sniffing [4].

According to a previous study on healthy volunteers, the normal pattern of diaphragm motion evaluated with DDR is characterized by higher speed and excursion of the left diaphragm (Figure 4) [10], with a mean excursion during forced deep breathing of 49 ± 17 mm on the right-hand side and 52 ± 16 mm on the left [10], and a smaller excursion in tidal breathing of 11 ± 4 mm on the right-hand side and 15 ± 5 mm on the left [11]. The same pattern was already observed in an ultrasound-based study [9].

**Figure 5:**
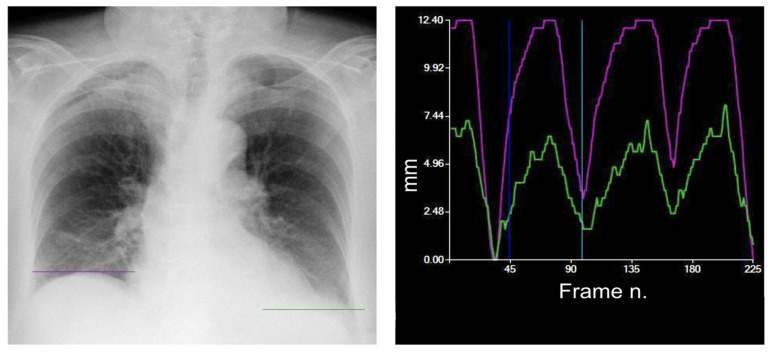
Reduced motility of the left diaphragm. Purple line = right diaphragm; green line = left diaphragm.

**Figure 6:**
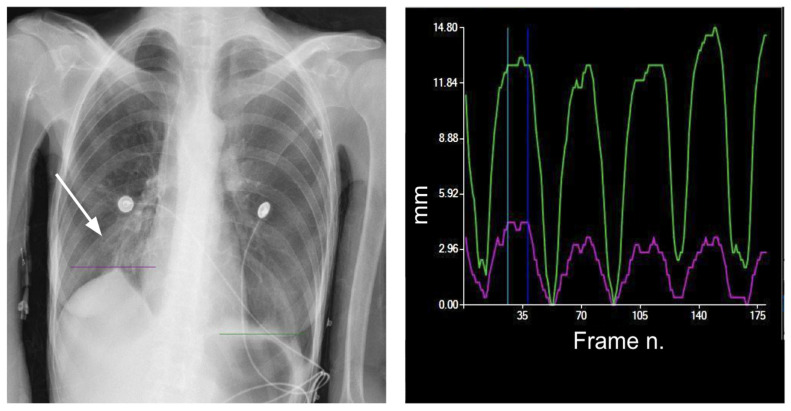
Impaired motility of the right diaphragm due to fibrotic alterations. Morphological evaluation of the X-ray exam shows an elevated and stapled right diaphragm with fibrotic bands in the lower right field (arrow). Purple line = right diaphragm; green line = left diaphragm.

**Figure 7:**
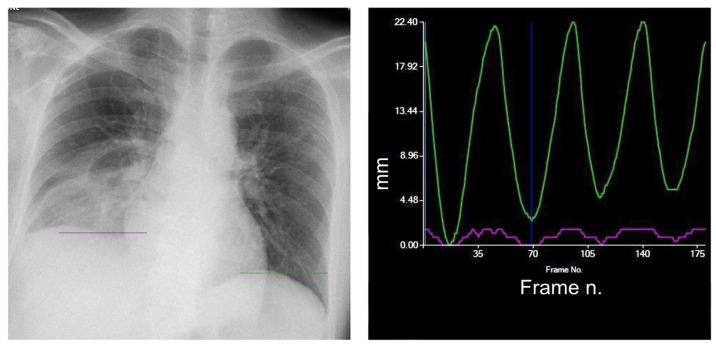
Right diaphragm palsy. The right diaphragm is characterized by a marked reduction in motion (purple curve). The motion of the left diaphragm (green curve) is normal. Purple line = right diaphragm; green line = left diaphragm.

Diaphragm-altered motility can be the result of different clinical conditions directly affecting the diaphragm, the rib cage, or the nerve pathways. These include vascular disease, post-traumatic lesions, infections, muscular and neuro-muscular diseases, neoplasms, and collagenous disorders [6]. Motility alterations of the diaphragm comprise hyper- or hypomobility (Figure 5 and Figure 6) up to paralysis (Figure 7).

This patient shows a reduction in the excursion of the left diaphragm (green curve), which is normally characterized by a curve higher than the one of the right diaphragm, whereas the right diaphragm (purple curve) moves normally over the breathing cycle.

Previous studies in standing acquisitions described significant differences in diaphragm motion between patients with chronic obstructive pulmonary disease (COPD) and healthy subjects [12]. Furthermore, conditions such as a high body mass index (BMI) and COPD proved to be associated with an increased diaphragm excursion: it was hypothesized that high-BMI patients required increased diaphragmatic motion to compensate for higher oxygen consumption, whereas, in COPD patients, increased diaphragm motion was interpreted as a compensation for abnormal gas lung exchanges [11].

**Figure 8:**
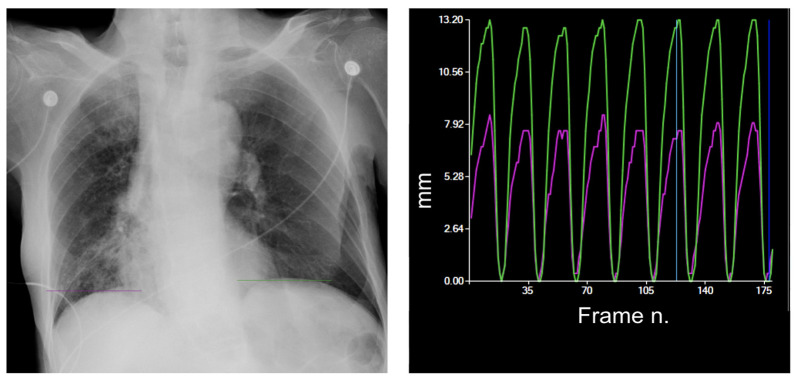
Increased respiratory rate. The study of diaphragmatic mobility allows you to easily calculate the respiratory rate, which is increased (32 cycles/minute) in a patient with respiratory distress. The diaphragmatic excursion pattern is normal, with a regular higher curve of the left diaphragm. Purple line = right diaphragm; green line = left diaphragm.

Furthermore, the study of diaphragm mobility patterns can provide information on the respiratory rate, which is frequently altered in hospitalized patients, particularly in emergency settings where respiratory distress and hyperventilation may be present (Figure 8).

The accessibility of bedside DDR images using portable X-ray technology facilitates the examination of diaphragm patterns and offers valuable information on respiratory rates and alterations that impact diaphragm motion. Moreover, the streamlined acquisition process eliminates the need for patient transportation, enhancing efficiency and a patient-centric approach in emergency departments.

## 4. Calculation of the Lung Area

**Figure 9:**
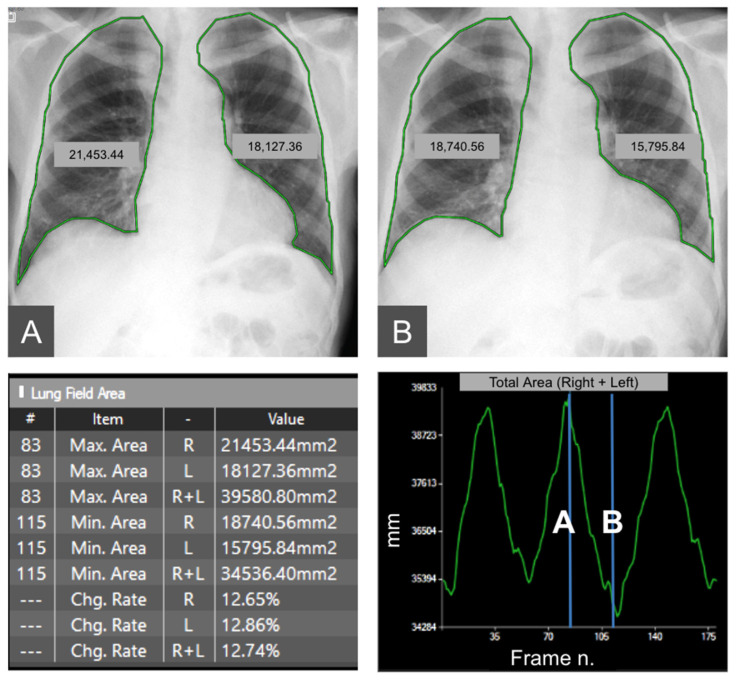
Automatic segmentation of projected lung area in maximum expiration (**A**) and inspiration (**B**). The areas are automatically calculated by the software, based on the highest (representing inspiration) and lowest peak (representing exspiration) of the curve of diaphragmatic motion. Moreover, the maximum and minimum areas of the right and left lungs and of both lungs are automatically provided in mm^2^ and as the area change rate in %.

From each DDR frame, the lung area, which is also called the projected lung area, can be automatically calculated using edge detection methods (e.g., Prewitt Filter) in the post-processing phase [13] (Figure 9). The variation in lung area is usually expressed in terms of the changing rate (%) using as a reference the minimum area during maximum expiration.

Inspiratory lung area has been found to correlate well with forced vital capacity (FVC) at spirogram in respiratory diseases [14,15] and in healthy volunteers [13].

Further correlations between DDR-obtained quantitative parameters and pulmonary function test results have been investigated, highlighting the potential added value that DDR may play in patients when spirometry testing is not available.

Projected lung area and Forced Expiratory Volume in the first second (FEV1) have a variable relationship. The inspiratory lung area correlates with FEV1 in both healthy people and patients with restrictive lung diseases [14,16]. This relationship, however, was not found in patients with COPD, where FEV1 is a key marker of disease severity [16]. Similarly, while changes in projected lung area had a moderate correlation with FEV1 in a group of people with cystic fibrosis bronchiectasis [15], it only had a weak correlation in people with COPD. These findings could be attributed to underlying pathophysiology (emphysema or air trapping, for example) or the small number of COPD patients enrolled in the studies.

In restrictive lung conditions, the inspiratory projected lung area was significantly reduced [16], whereas in severe obstructive lung disorders, the inspiratory lung area significantly increased compared with healthy controls, which is a sign of underlying air trapping [17].

The changing rate in the projected lung area during the respiratory cycle is related to ventilation. In our clinical experience, the changing rate in the lung area turned out to be smaller in patients showing a marked reduction in diaphragmatic excursion and greater in patients who hyperventilate due to respiratory distress. Further studies on bedridden patients or those with respiratory distress are needed for the quantitative evaluation of the relationship between variation in lung area, ventilation, and respiratory tests and to clarify the impact of these imaging-derived data in the management of complex patients.

## 5. Lung Movement

**Figure 10:**
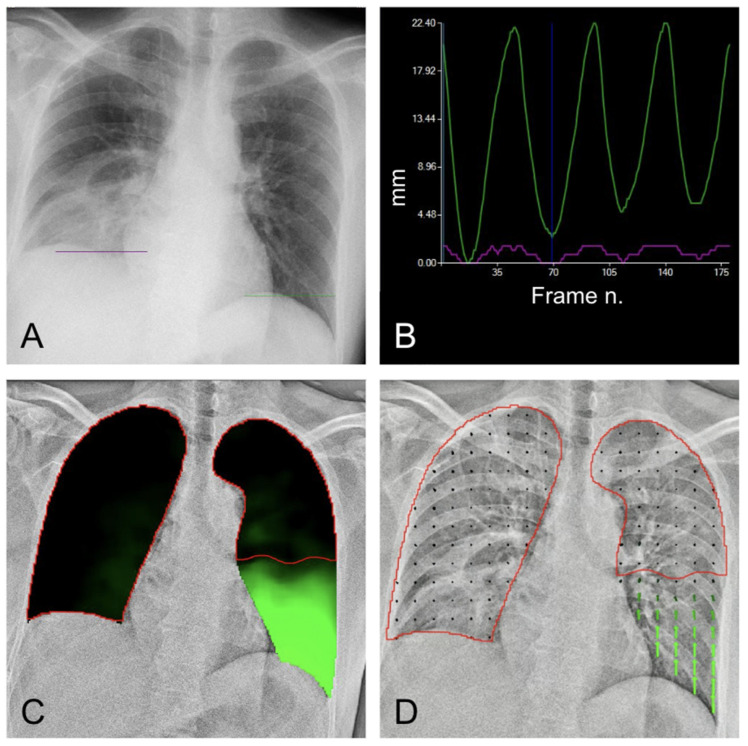
Lung motion analysis. (**A**) Morphological evaluation of radiographic examination shows elevated right hemi diaphragm and fibrotic bands in the right lower lung field. (**B**) DM mode shows reduced motility of the right diaphragm. Purple line = right diaphragm; green line = left diaphragm. (**C**) LM analysis shows poor ventilation of the right lung compared with the left side. (**D**) Vector maps highlight the lack of mobility in the lower right base (no detectable arrows = no movement).

Diaphragm movement is closely linked to lung movement. Useful information regarding the presence of pleural adhesions may be derived from the lung motion analysis map (LM-mode) [18], where green areas represent lung areas characterized by normal motility, whereas black regions have markedly reduced or no motion (Figure 10).

Sequential images with the diaphragm moving upward were selected to measure the motion vector of lung structures in the expiratory phase. Dense Optical Flow by Gunnar FarneBack technique was used to estimate pixel-by-pixel motion (optical flow) between two consecutive frames in each local lung area on bone-suppressed images. Optical flow was calculated as an array of flow vectors in terms of magnitude and direction. The total amount of movement was calculated by connecting the movement between two consecutive frames. An open-source computer vision library (OpenCV 3.4.3) was used. The resulting output is a two-dimensional vector field, where each vector is a displacement vector that shows the movement of points from a reference frame to the current frame (Figure 10D, Supplementary Material Video S2).

The assessment of the presence of pleural adhesion is important in the diagnosis and preoperatory planning of lung cancer [19].

In a study by Tanaka et al. the authors evaluated the utility of dynamic chest radiography (DCR) motion analysis for preoperative evaluation of pleural adhesions in lung cancer patients. The research involved 146 patients, with 25 having pleural adhesions. By measuring local motion vectors and calculating the percentage of lung area with poor motion, the study established a threshold of ≥49.0% to indicate pleural adhesions. The DCR-based motion analysis demonstrated 84.0% sensitivity, 61.2% specificity, 30.9% positive predictive value (PPV), and 94.9% negative predictive value (NPV). Although the method cannot pinpoint the exact location of adhesions, it provides valuable information for preliminary surgical planning [18].

## 6. Lung Ventilation

**Figure 11:**
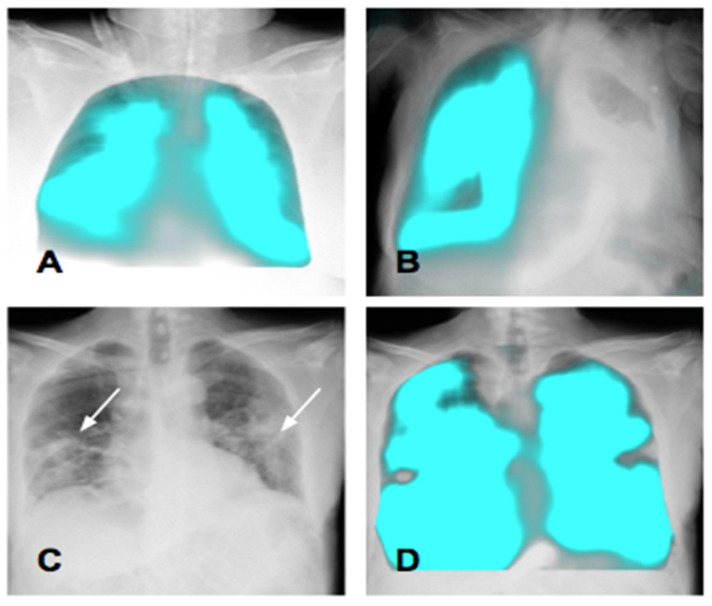
Ventilation. (**A**) Ventilation analysis in a bedridden patient in the Intensive Care Unit. The blue areas allow for the visualization of the ventilated lung areas, demonstrating good ventilation of the pulmonary fields. (**B**) Ventilation map in a patient with huge left pleural effusion: the right lung shows regular ventilation, whereas no ventilation is visible on the left side. (**C**,**D**) The ventilation map confirms the presence of hypoventilated areas corresponding to pulmonary consolidations (arrows).

Preliminary studies have established that DDR can detect changes in pixel density and related signal characteristics over time and can be applied to detect regional differences in ventilation [20,21]. In the PL-mode (Figure 11), the visualization of the signal change in the lung tissue behavior is realized by extracting the periodic signal change related to respiration from the moving image.

Lung translucency varies according to different conditions and constantly throughout respiration. During inspiration, the air volume inside the alveoli, bronchiole, and bronchi increases, resulting in a decrease in pixel values on X-ray and an increase in translucency, whereas during expiration, the air volume decreases, resulting in an increase in pixel value and a decrease in translucency. The difference in pixel translucency values between the maximum inspiration and expiration frames reflects the extent of ventilation. The distribution of these functional values could be visualized in color maps (Figure 11A).

Dynamic image registration is used for pixel value analysis during breathing to match the corresponding anatomic areas on sequential X-ray images. The determination of the respiratory phase is based on diaphragm cinematic analysis: the frames with the lowest and highest peaks of the diaphragm kinetics curve are used as references for the maximum inspiratory and expiratory frames, respectively. Inter-frame variations in the pixel values are considered to represent pulmonary airflow at each time point in each local area [4,21].

Signal value change maps generated by the PL-mode can be consulted alongside static images to confirm the presence of local changes related to respiration in the lung fields (Figure 11C,D). From a clinical perspective, the most promising application could be the monitoring of the effectiveness of therapy with the detection of regional differences in ventilation at different time points.

## 7. Lung Perfusion Assessment

DDR has been explored to detect regional changes in pulmonary perfusion by analyzing pixel density and related signal properties over time [1,4,5,22].

The advantages of DDR compared with other imaging techniques include the elimination of the need for contrast media or radionuclides administration, the ability to assess pulmonary perfusion in patients with impaired renal function and allergies to iodinated contrast agents, and a reduction in examination costs [1,4,5].

DDR imaging is a rapid and possibly widespread imaging technique that requires only 6 s of apnea for capture and less than one minute for post-processing analysis. The availability of portable equipment allows its acquisition also in the Emergency Department, without the need for moving critical patients to CT suites: this eases the diagnostic process in emergency medicine and strengthens the diagnostic power.

Different methods can be used to obtain perfusion maps [4].

**Figure 12:**
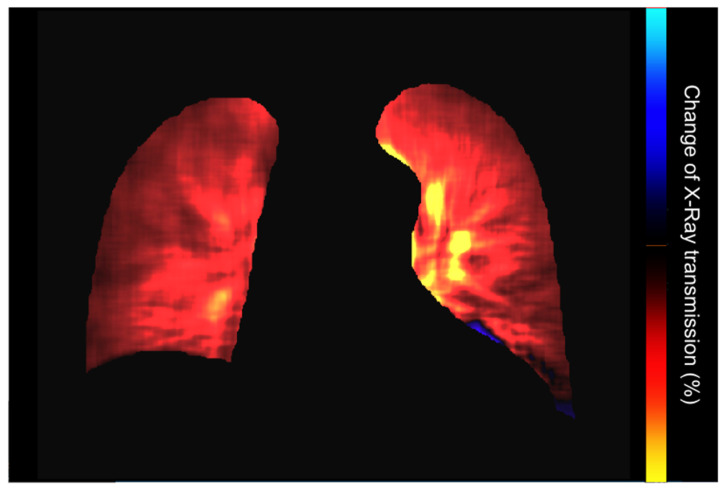
Normal perfusion map.

A map shows the pixel value change rate (calculated from interframe discrepancies). The PH2-mode uses the reference frame subtraction method to visualize the degree of pixel value change from the baseline timing (end-diastolic phase). After removing the respiratory-related temporal changes in pixel values using a band-pass filter, the interval changes from the reference frame (end-diastolic phase) at each phase are analyzed. A large interval change in the pixel value is colored red and yellow [4,5] (Figure 12).

Preliminary assessment of DDR perfusion images is necessary in order to avoid misdiagnosis of pulmonary perfusion abnormalities related to nonvascular alterations or post-processing related issues [23]. Standard static or dynamic chest X-rays should be carefully evaluated to identify potential lung abnormalities (mass/nodule, opacity, reticulation, bulla, pneumothorax, and pleural effusion) that can confound interpretation. After that, dynamic lung perfusion images and the map are assessed to detect perfusion abnormalities. The findings are reported as pathological when the alterations in the dynamic perfusion images or lung perfusion map are combined with a normal lung appearance in the corresponding areas in the dynamic or static images, thus suggesting the existence of a perfusion defect [1,4,5].

DDR can be used also in chronic conditions (chronic thromboembolic pulmonary hypertension (CTEPH)), as well as in the emergency acute setting (acute pulmonary embolism).

CTEPH is the result of persistent pulmonary thromboembolism with chronic obstruction of pulmonary vessels, progressive pulmonary artery remodeling, and pulmonary hypertension [24]. In the cases of missed or delayed diagnoses, the patient’s prognosis is poor due to subsequent right heart failure [25]. Early detection and treatment with pulmonary endarterectomy, pulmonary vasodilation, and balloon pulmonary angioplasty improves both pulmonary and clinical results [26].

Ventilation–perfusion scintigraphy is the gold standard for diagnosing CTEPH thanks to its high accuracy [27], but its availability is limited in clinical practice by the large machinery needed and experienced personnel. Therefore, a method for diagnosing CTEPH with higher availability and less radiation exposure is desirable to enable early detection and treatment.

In the presence of pulmonary perfusion defects, DDR exhibits triangular or wedge-shaped deficiencies, similar to perfusion scintigraphy and invasive pulmonary angiography [28,29]. In a recent small trial, DDR showed efficacy comparable to that of a ventilation/perfusion scan in detecting CTEPH [30], but a prospective validation study with a larger sample size is required to validate DDR as an effective alternative to a ventilation/perfusion scan.

Acute pulmonary embolism is a potentially life-threatening condition caused by sudden obstruction of blood flow in a pulmonary artery leading to reduced perfusion and damage to lung tissue. The incidence is between 60 and 120 per 100,000 per year [31]. It should be suspected in patients with acute chest pain, shortness of breath, or syncope, and its diagnosis includes pre-test clinical probability assessment, D-dimer testing, and chest imaging [32].

CT pulmonary angiography represents the gold standard imaging technique for the diagnosis [31], but the administration of iodinated contrast media may be problematic in patients with altered renal function or contrast allergies.

DDR could be a helpful tool in such cases. From the available data, DDR sensitivity of acute pulmonary embolism diagnosis is lower than that of CT angiography, and a comprehensive approach combined with D-dimer testing and clinical pre-test probability is recommended [33,34].

Acute pulmonary embolism on DDR is characterized by triangular or wedge-shaped perfusion defects, originating from segmental or lobar perfusion alterations.

Other vascular causes of segmental hypoperfusion include vascular compression/compromise from the tumor or fibrosing mediastinitis, vasculitis affecting the pulmonary vessels [35], altered pulmonary circulation due to pulmonary artery hypoplasia or pulmonary sequestration, and granulomatous disease affecting the vessels, such as pulmonary sarcoidosis.

DDR can offer several advantages for perfusion assessment as it is a non-invasive method that does not require contrast media or radionuclides, resulting in almost no contraindications. It is a rapid and readily available imaging technique that can be performed directly on a patient’s bedside and can avoid patient transportation for limited patient mobility with a quick capture time of 6 s and 1 min for post-processing analysis. This efficiency might be particularly beneficial in emergency medicine and Intensive Care Units, facilitating prompt diagnostic assessments. Moreover, portable devices are cost-effective systems and more accessible if compared with other modalities like CT, MRI, and Scintigraphy. DDR radiation doses are significantly lower than lung ventilation/perfusion (V/Q) scanning and standard CT pulmonary angiography (CTPA). This makes DDR a safer option, with doses around 0.2 mSv compared with 2 mSv for V/Q scan and 4–6 mSv for CTPA.

However, further studies are needed to precisely establish which patients are eligible for a DDR study as an alternative to CT.

## 8. Post-Procedural Follow-Up

A chest X-ray is the recommended imaging technique to monitor the occurrence of post-procedural pneumothorax (percutaneous transthoracic needle biopsy, central venous catheter placement, etc.) [36,37].

**Figure 13:**
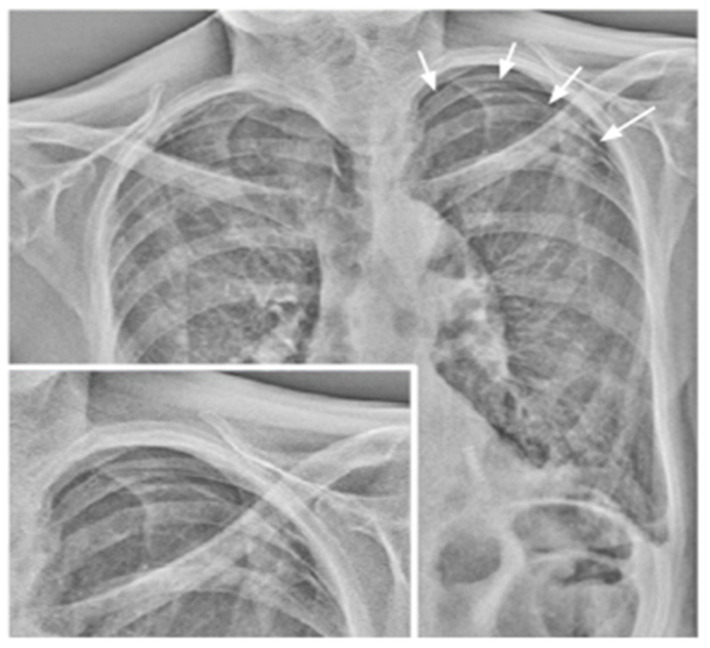
Post-procedural follow-up. The frequency-enhanced mode can improve soft tissue highlighting some findings such as a small post-biopsy left apical pneumothorax (white arrows).

The availability of a dynamic X-ray acquisition showing both the inspiratory and expiratory phases may increase the diagnostic accuracy for pneumothorax in bedridden patients in its detection could be challenging. In such cases, FE mode provides a useful tool (Figure 13).

## 9. Challenges

DDR is a new X-ray technique and, therefore, further dose and radioprotection assessment must be performed to optimize radiation exposure and check the safety distance between patients hospitalized in the same room.

**Figure 14:**
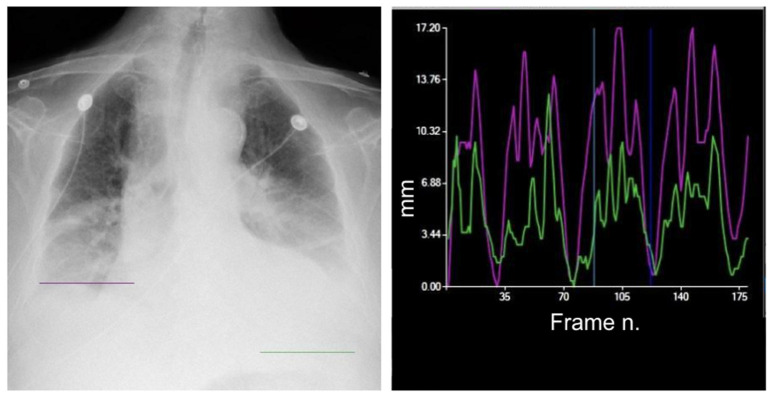
In the case of pulmonary consolidation or pleural effusion, the diaphragm can be non-recognizable, and the tracking can be imprecise, as for the left diaphragm in this case. Purple line = right diaphragm; green line = left diaphragm.

According to clinical request, the radiologist must always provide precise indications on how the acquisition must be performed (breathing patterns or breath holding) and carry out a preliminary assessment of the diagnostic quality of the acquisition. Furthermore, post-processing should be evaluated to detect possible errors and pitfalls that should be corrected (Figure 14), considering that in-bed patients present much more challenging conditions for post-processing models than standing patients or healthy volunteers.

Although several publications have highlighted the advantages of DDR over the standard technique, this technology has recently been introduced into portable radiographic systems. Therefore, further investigations using dedicated clinical studies are necessary both in orthostatic and bedridden patients. In addition, the clinical advantages of modalities such as BS-mode and FE-mode are currently still little explored in the study of the chest.

Enhancing the comprehension of DDR capabilities will be further enriched using comparative analyses with other modalities, such as Computed Tomography [8]. This type of investigation will contribute to a further understanding of the advantages and applications of this new technology, comparing results with established and widely used technologies.

## 10. Future Directions

The future holds promising advancements in dynamic digital radiography (DDR), which is driven toward enhanced capabilities and diagnostic precision.

On the hardware front, there is a growing focus on refining imaging devices and sensor technologies. Advancements in high-speed, high-resolution detectors will enable DDR systems to capture dynamic anatomical movements with unprecedented clarity and detail. Additionally, innovations in dual-energy methods and the integration of novel materials may enhance the versatility and diagnostic potential of DDR, offering insights into tissue composition and functional characteristics [38,39]. In terms of software, the integration of artificial intelligence (AI) and deep learning algorithms is expected to play a pivotal role in DDR’s evolution. Computer-aided diagnosis (CAD) schemes play a crucial role in interpreting DDR findings, given the complexity of information that can be challenging to interpret solely with visual assessment [8]. Examining chest wall details, such as rib kinematics, may augment the diagnostic value of DDR. AI-powered image processing and analysis tools can automate the detection of subtle abnormalities, providing radiologists with efficient and accurate diagnostic support [40]. These algorithms may also contribute to real-time image enhancement, reducing the need for extensive post-processing and expediting the interpretation of dynamic radiographic reformats. While the diaphragmatic motion studies discussed in this article focused solely on the highest points of the diaphragms, it is feasible to assess the motion of the entire diaphragm. Additionally, non-rigid image registration on DXR images allows for the observation of chest wall and lung field motion. Furthermore, these applications may extend beyond the lungs and diaphragm, proving useful for dynamically evaluating the spine, joints, muscles, bones, or heart and great vessels. Such assessments have the potential to offer additional diagnostic value in different clinical settings.

As DDR continues to evolve, it may extend its applications beyond traditional chest and musculoskeletal examination studies to dynamic evaluations of cardiac function, gastrointestinal motility, and respiratory dynamics. This expansion of capabilities could open new avenues for comprehensive and non-invasive assessments of various physiological systems, providing valuable clinical insights.

## 11. Conclusions

DDR is a functional imaging technique that offers helpful data for the assessment of lung perfusion, ventilation, diaphragmatic motion, and improvement in lung abnormalities assessment. It provides consecutive sequential images produced by pulsating X-ray emission. The acquired images are then processed, and the results are visualized, using computerized image processing techniques. Compared with alternative imaging techniques, DDR exhibits several benefits including portability, flexibility, accessibility, patient-centric focus, and user-friendliness. The recent release of DDR technology on portable devices further enhances the flexibility of this technology on bedridden patients and represents significant potential, especially as a new portable technique capable of surpassing the diagnostic limitations of traditional static X-ray portable systems. Further clinical studies including a comparison with other diagnostic modalities are mandatory for the validation of diagnostic performances of this technology. The implementation and development of a new post-processing function may improve quantitative analysis expanding its potential application beyond thoracic imaging.

## Figures and Tables

**Table 1:** illustrates the different post-processing modalities, their mechanisms, and potential clinical applications.

Modality	Function	Mechanism	Potential Clinical Applications
DM-mode	Tracking diaphragm motion. Automatic calculation of the lung area and the relative changing rate (%).	Tracking of diaphragmatic domes represented in a motion–time graph. Area detection through an edge detection method in the post processing phase.	Diagnosis of diaphragm motion impairment and palsy. Automatic calculation of the respiratory rate. Analysis of lung dynamics in patients with restrictive/obstructive diseases. Follow-up of patients in pulmonary rehabilitation.
LM-mode	Vector field representing the movement of different lung areas.	Pixel-by-pixel analysis and tracking in consecutive frames resulting in a two-dimensional vector field. Uses BS-images.	Differential motion analysis of different lung areas; useful for the detection of adhesions and preoperative planning.
PL-mode	Color-coded map of the lung ventilation in different areas.	Analysis of the pixel values variations during the breath cycle.	Detection of regional differences in ventilation. Useful for the follow-up of therap, especially in Intensive Care Units.
FE-mode	Improve visualization of soft-tissues density components.	Post-processing enhancing high-spatial frequencies.	Better visualization of soft tissue alterations.
BS-mode	Bone suppression.	Signal attenuation of costal and clavicular bones within the lung field. Preliminary to LM analysis.	Better visualization of tissues other than bone, with better detection of lung nodules and consolidations.
PH2-mode	Color-coded map representing lung perfusion.	Analysis of pixel value changes from the baseline timing (end diastolic phase).	Analysis of the pulmonary perfusion, diagnosis of pulmonary embolism.

PL = (reference frame ratio circulation) = “pixel value change—low-frequency processing”, FE = frequency enhanced, BS = bone suppression, LM = lung motion, PH2 = “pixel value change—high-frequency processing”, DM = diaphragm motion.

## Data Availability

Not applicable.

## Supplementary Materials

The following supporting information can be downloaded at: https://www.mdpi.com/article/10.3390/medsci12010010/s1, Video S1: DM-mode showing impaired right diaphragm motility. Video S2: LM-mode showing lack of mobility in the lower right base.
